# Prevalence and Clinical Implications of Pulmonary Vein Stenosis in Bronchiectasis: A 3D Reconstruction CT Study

**DOI:** 10.3390/arm92060046

**Published:** 2024-12-16

**Authors:** Xin Li, Yang Gu, Jinbai Miao, Ying Ji, Mingming Shao, Bin Hu

**Affiliations:** 1 Department of Thoracic Surgery, Beijing Institute of Respiratory Medicine and Beijing Chao-Yang Hospital, Capital Medical University, Beijing 100020, China; lx_illusion@126.com (X.L.); guyang1998@163.com (Y.G.); miaojinbai@163.com (J.M.); 15675112499@163.com (Y.J.); 2 Department of Respiratory and Critical Care Medicine, Beijing Institute of Respiratory Medicine and Beijing Chao-Yang Hospital, Capital Medical University, Beijing 100020, China

**Keywords:** bronchiectasis, pulmonary venous stenosis, 3D reconstruction, lung volume, lung function

## Abstract

**Highlights:**

**What are the main findings?**

- Significant prevalence of pulmonary vein stenosis (PVS) in bronchiectasis patients, particularly in the left lower lobe.
- Pulmonary vein stenosis is associated with disease progression, reduced lung volume, and impaired lung function in bronchiectasis patients.

**What is the implication of the main finding?**

- Pulmonary vein stenosis in bronchiectasis has been identified and statistically analyzed for the first time, providing a new direction for future research on bronchiectasis.
- Pulmonary vein stenosis may be an important factor in the progression of bronchiectasis and a potential therapeutic target.

**Abstract:**

Background: Recent studies on bronchiectasis have revealed significant structural abnormalities and pathophysiological changes. However, there is limited research focused on pulmonary venous variability and congenital variation. Through our surgical observations, we noted that coarctation of pulmonary veins and atrophied lung volume are relatively common in bronchiectasis patients. Therefore, we conducted a retrospective study to explore pulmonary venous variation and secondary manifestations in bronchiectasis cases, utilizing 3D reconstruction software (Mimics Innovation Suite 21.0, Materialise Dental, Leuven, Belgium) to draw conclusions supported by statistical evidence. Method: This retrospective study included patients with bronchiectasis and healthy individuals who underwent CT examinations at Beijing Chao-Yang Hospital between January 2017 and July 2023. Chest CT data were reconstructed using Materialise Mimics. Pulmonary veins and lung lobes were segmented from surrounding tissue based on an appropriate threshold determined by local grey values and image gradients. Subsequently, venous cross-sectional areas and lung volumes were measured for statistical analysis. Result: CT data from 174 inpatients with bronchiectasis and 75 cases from the health examination center were included. Three-dimensional reconstruction data revealed a significant reduction in cross-sectional areas of pulmonary veins in the left lower lobe (*p* < 0.001), the right lower lobe (*p* = 0.030), and the right middle lobe (*p* = 0.009) of bronchiectasis patients. Subgroup analyses indicated that approximately 73.5% of localized cases of the left lower lobe exhibited pulmonary vein stenosis, while in the diffuse group, this proportion was only 52.6%. Furthermore, the cross-sectional area of pulmonary veins had a gradually decreasing trend, based on a small sample. Lung function tests showed significant reductions in FEV1, FVC, and FEV1% in bronchiectasis patients, attributed to the loss of lung volume in the left lower lobe, which accounted for 60.9% of the included sample. Conclusions: Our recent findings suggest that pulmonary venous stenosis is a common variation in bronchiectasis and is often observed concurrently with reduced lung volume, particularly affecting the left lower lobe. Moreover, localized cases are more likely to suffer from pulmonary venous stenosis, with an ambiguous downtrend as the disease progresses. In conclusion, increased attention to pulmonary venous variation in bronchiectasis is warranted, and exploring new therapies to intervene in the early stages or alleviate obstruction may be beneficial.

## 1. Introduction

Bronchiectasis stands as one of the most prevalent respiratory diseases globally, with recent data reporting an incidence ranging from 67 to 1200 cases per 100,000 individuals [1]. Bronchiectasis has been demonstrated to be associated with long-term chronic infection and lead to dilatated bronchial luminal, thickened bronchial walls, and varying degrees of airway obstruction [2,3]. As the most common clinical symptoms, cough, sputum production, and hemoptysis significantly impact the quality of life for afflicted individuals [4]. Recent studies have unveiled several structural abnormalities in bronchiectasis patients, including hyperplasia and dilatation of bronchial arteries, thickened pulmonary artery walls, dysplasia of ipsilateral pulmonary arteries, and reduction of ipsilateral lung volume [5,6,7,8]. However, there remains a paucity of research focusing on pulmonary venous variability in bronchiectasis patients.

Pulmonary vein stenosis (PVS) is an uncommon condition that, if left untreated, can lead to substantial morbidity and mortality. PVS is marked by the progressive narrowing of one or more pulmonary vein lumens. As hemodynamic alterations become significant and vascular resistance rises, patients may experience clinical symptoms such as dyspnea, chest pain, cough, and hemoptysis [9,10]. PVS is categorized into congenital pulmonary vein stenosis and acquired pulmonary vein stenosis [10]. Congenital PVS frequently occurs in association with congenital heart defects. Acquired PVS, on the other hand, is typically induced by factors such as catheter ablation injury from atrial fibrillation treatment, fibrosing mediastinitis, sarcoidosis, tumor compression, anomalous pulmonary venous return repair, and lung transplantation [9,11,12,13,14,15,16]. In recent years, advancements in imaging technology have enabled more precise diagnosis and assessment of PVS. However, the incidence and clinical significance of PVS in patients with bronchiectasis have not yet been thoroughly investigated.

Our observations during bronchiectasis surgery reveal that coarctation of pulmonary veins and atrophied lung volume are relatively common occurrences. Previous reviews have indicated that pulmonary venous stenosis can precipitate obstructions in pulmonary and bronchial circulation, redistribute pulmonary arterial flow, and even lead to pulmonary venous hypertension. Additionally, remodeling and reduced branching of pulmonary arteries have been noted in severe cases. Consequently, patients frequently report a variety of clinical symptoms such as cough, dyspnea, hemoptysis, and recurrent lung infections [17,18,19,20]. However, previous reports have indicated that in children with PVS, bronchial wall thickening and bronchiectasis have not been observed [21,22]. Moreover, there are currently no studies investigating the association between bronchiectasis in adults and PVS.

This retrospective study was designed to explore pulmonary venous variation in bronchiectasis through three-dimensional reconstruction utilizing software, thereby drawing conclusions supported by statistical evidence.

## 2. Methods

### 2.1. Study Population and Design

High-resolution CT serves as the gold standard for diagnosis, offering superior visualization of classical airway dilatation and bronchial wall thickening (resembling a tram-track) in bronchiectasis. Enlarged airways often exhibit a signet-ring sign in cross section with their accompanying pulmonary artery. Additionally, cysts may be discerned. For this retrospective study, patients diagnosed with bronchiectasis through chest CT examinations at Beijing Chao-Yang Hospital between January 2017 and July 2023 were included. CT data from a healthy population undergoing physical examinations at the same facility during the specified period served as the control group. Approval for the study was obtained from the Institutional Review Board of our center, and the requirement for informed consent was waived due to the retrospective nature of the study.

The inclusion criteria for this study were: (1) age ≥ 18 years; (2) complete clinical data; (3) chest CT performed at our center; (4) absence of a history of thoracic surgery; and (5) primary diagnosis is bronchiectasis, with or without hemoptysis and infection. Exclusion criteria were: (1) unclear 3D reconstruction model for measurements; (2) combination of COPD, bronchial asthma, bacterial pneumonia, interstitial pneumonia, pulmonary atelectasis, pulmonary fungal infection, or respiratory failure; or (3) history of pulmonary surgery.

### 2.2. The Definitions for Study Variables and Outcomes

Pulmonary vein cross-sectional area (PVCA) is defined as the measured area of pulmonary veins (in mm^2^) captured 5 mm from the veno-atrial junction to assess for stenosis.

PVS is defined by a reduction in PVCA to below 75% of the normal mean.

### 2.3. Three-Dimensional Reconstruction

The chest CT data were exported in DICOM format and subsequently imported into Materialise Mimics 21.0 (Mimics Innovation Suite 21.0, Materialise Dental, Leuven, Belgium). The target pulmonary veins were segmented from the surrounding tissue by applying an appropriate threshold based on the local grey value and image gradient. Specifically, the minimum threshold was set at −50 Hounsfield units (HU) for chest CT scans and +100 HU for contrast-enhanced CT scans. Finally, the cross-sectional area of the pulmonary veins was intercepted and measured at a distance of 5 mm from the veno-atrial junction.

### 2.4. Statistical Methods

All statistical analyses were performed using IBM SPSS Version 26.0 software and R version 4.0.3. Normally distributed variables were presented as the mean ± standard deviation (M ± SD), and comparisons between two groups were made using the independent samples t-test. Non-normally distributed variables were expressed as median (range) and analyzed using the Mann–Whitney U test. Categorical variables were expressed as numbers (percentages) and analyzed using either the chi-square test or Fisher’s exact test. Two-sided *p* < 0.05 was considered statistically significant.

## 3. Results

### 3.1. Baseline Characteristics

**Figure 1:**
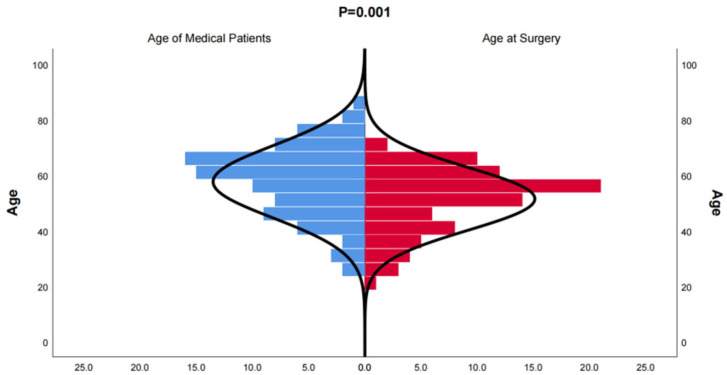
Distribution of onset age of medical patients diagnosed with bronchiectasis and age at the time of surgery for bronchiectasis. The age distribution of patients who underwent surgical treatment for bronchiectasis is younger than that of patients who received conservative treatment.

**Figure 2:**
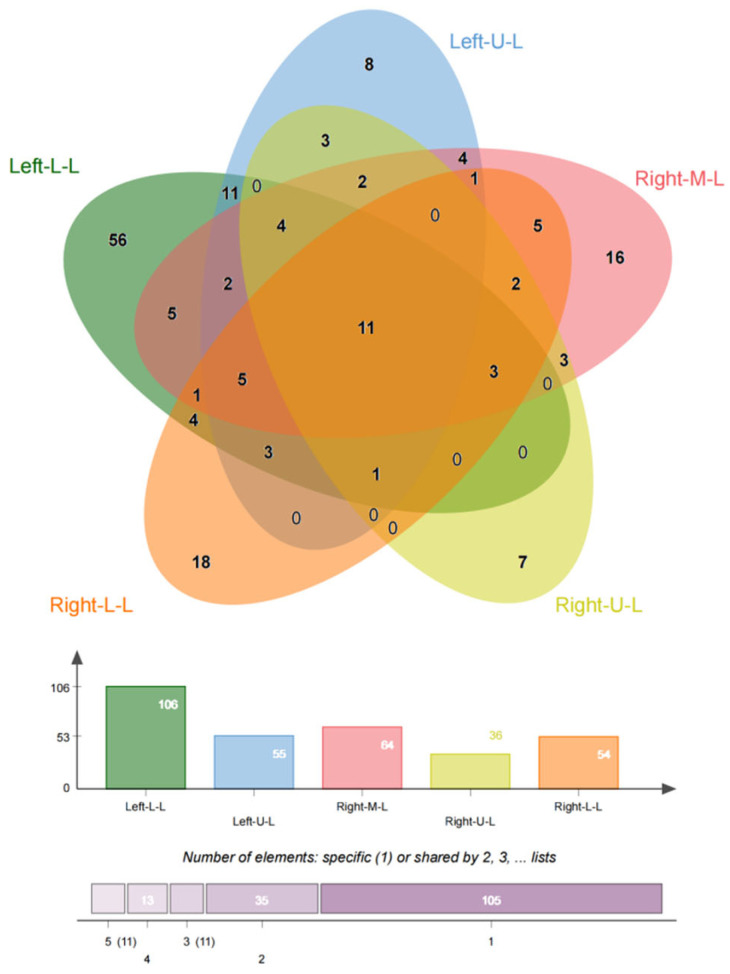
Location of bronchiectasis. The PVS is more common in the left lower lobe, right middle lobe, and right lower lobe, even though in most cases the lesions involve multiple lobes.

In this study, we included 174 inpatients diagnosed with bronchiectasis and 75 individuals from a health examination center. Subsequently, the CT data were reconstructed into 3D models using Mimics Medical 21.0 (Table 1). Our baseline data revealed that the onset age of medical patients ranged from 21 to 85 years old, with some patients experiencing deterioration and undergoing surgery between the ages of 21 and 70 (Figure 1). There was no significant difference in the incidence between males and females, and smoking was not identified as a risk factor for bronchiectasis. However, the lung function of healthy individuals was significantly better than that of individuals with bronchiectasis. Moreover, in line with previous studies, the majority of bronchiectasis cases involved the left lower lobe (106 cases, 60.9%) and the right middle lobe (64 cases, 36.8%). Additionally, most cases of left lower lobe bronchiectasis were accompanied by varying degrees of lingual lesions (38/106 cases,35.9%) (Figure 2).

### 3.2. Pulmonary Venous Stenosis Found in the Bilateral Lower Lobes and Middle Lobe of Patients with Bronchiectasis

**Figure 3:**
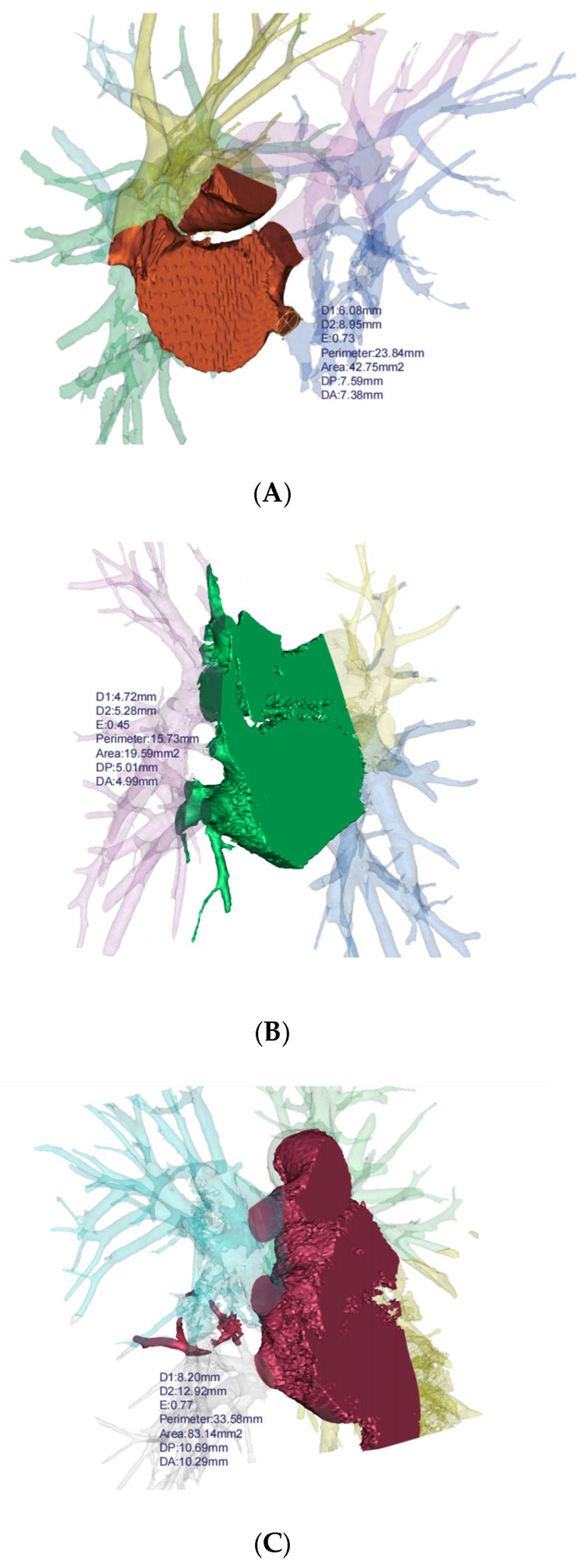
3D reconstruction for bronchiectasis of bilateral lower lobes and middle lobe. (**A**) PVS in left lower lobe, (**B**) PVS in right middle lobe, (**C**) PVS in right lower lobe. D1: diameter along major axis, D2: diameter along minor axis, E: eccentricity, DP: distance to periapsis, DA: distance to apoapsis.

Based on the 3D reconstruction data, the cross-sectional areas (measured 5 mm from the veno-atrial junction) of pulmonary veins were measured and compared between patients with bronchiectasis and healthy individuals for each lung lobe (Figure 3). The analysis revealed a reduction in cross-sectional areas in the left lower lobe (*p* < 0.001), the right lower lobe (*p* = 0.030), and the middle lobe (*p* = 0.009) of bronchiectasis patients. Particularly noteworthy is the observation of more severe stenosis or even occlusion in the pulmonary veins of the left lower lobe (cross-sectional area 83.08 mm^2^ vs. 123.99 mm^2^), indicating its high prevalence as a location of pathology (Table 2). In addition, the lung volume (*p* = 0.003) and volume ratio (*p* < 0.001) of bronchiectasis patients with lesions located in the left lower lobe are significantly smaller than those in the healthy population.

Furthermore, the above cases were stratified into two groups: (1) localized, involving only one lobe in bronchiectasis, and (2) diffuse, with bronchiectasis present in two or more lung lobes. Subgroup analyses demonstrated that approximately 73.5% of localized cases involving the left lower lobe exhibited pulmonary vein stenosis, defined as a cross-sectional area below 75% of the normal mean. In contrast, the proportion of cases with PVS in the diffuse group was only 52.6% (Table 3). In addition, the results also suggested a high proportion of PVS cases in the right lower lobe (5/11 cases,45.5%) and middle lobe (10/14 cases, 71.4%), but no statistical difference among different groups.

### 3.3. Trend of Gradual Aggravation of Pulmonary Venous Stenosis in a Long-Term Observation

**Figure 4:**
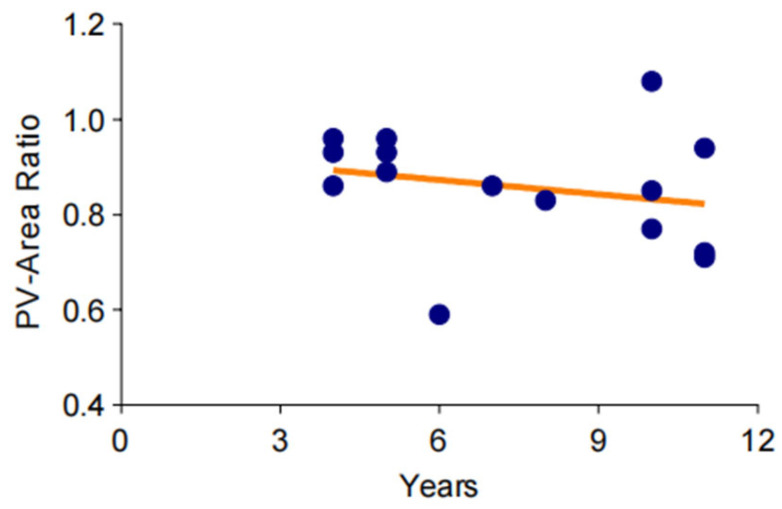
Trend of gradual aggravation of pulmonary venous stenosis in a long-term observation. The degree of pulmonary vein stenosis increases very slowly over time, and this change may take more than a decade or even longer.

To investigate the changes of pulmonary vein stenosis in the long-term chronic course of bronchiectasis, CT data from 12 patients over an 11-year period were standardized and analyzed. The results indicated that the cross-sectional area of the pulmonary veins showed an ambiguous downtrend over time (Figure 4).

### 3.4. Reduced Lung Function in Bronchiectasis, Owing to Left Lower Lobe Cases

**Figure 5:**
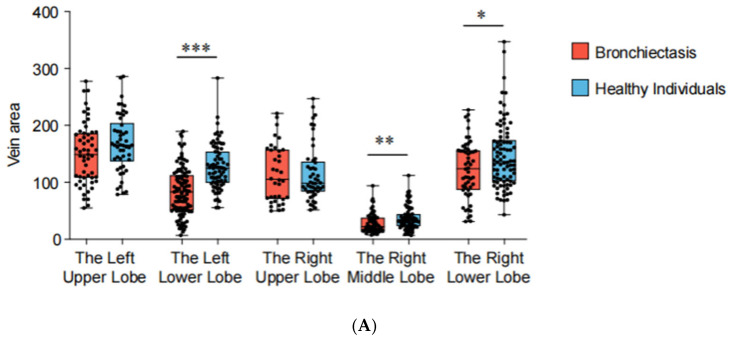

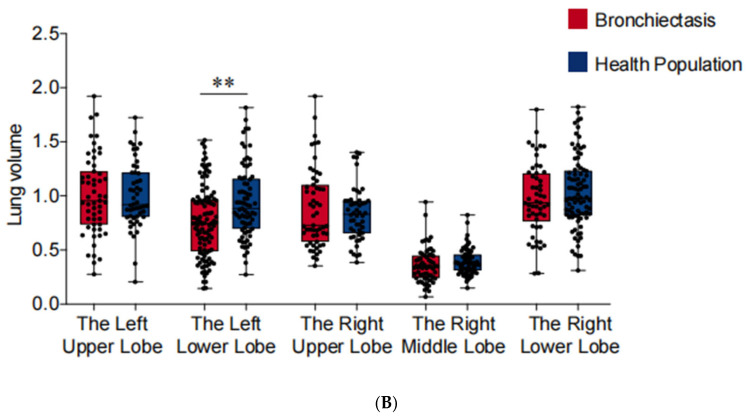
Pulmonary vein cross-sectional areas (PVCA) and lung lobe volumes of each lung lobe. *** *p* < 0.001, ** *p* < 0.01, * *p* < 0.05. (**A**) PVCA of each lung lobe. (**B**) Lung lobe volumes of each lung lobe.

In comparison to healthy individuals, lung function tests indicated a significant reduction in FEV1, FVC, and FEV1/FVC among patients with bronchiectasis (*p* < 0.05) (Table 1). Utilizing the 3D reconstruction technique, we established and measured the volume of each lung lobe. As shown in Figure 5, the loss of lung volume in cases involving the left lower lobe may be the primary cause of the aforementioned manifestations (*p* < 0.01). Furthermore, lung function and volume remained unchanged when other lung lobes were affected. Moreover, we observed a correlation between pulmonary dysfunction and volume reduction with pulmonary venous stenosis in cases of bronchiectasis involving the left lower lobe (Table 4).

## 4. Discussion

Most previous studies on bronchiectasis have primarily focused on anatomical changes in the pulmonary artery and bronchus, with diagnostic criteria often relying on the ratio of the bronchial diameter to the accompanying bronchial artery diameter [23,24]. The primary research directions regarding the pathogenesis of bronchiectasis include microbiological inflammation and inherited diseases. Pseudomonas aeruginosa is considered the most common bacterium detected in bronchiectasis, with its virulence playing a role in shaping host inflammatory and immune responses in the airway [25]. The persistence of bacterial pathogens triggers an inflammatory response leading to injury and abnormal remodeling of the airways, culminating in bronchiectasis [26]. However, existing research fails to explain why most bronchiectasis lesions are localized.

The majority of included cases undergoing surgery for bronchiectasis could be attributed to hemoptysis, purulent sputum production, and intolerable cough. Our findings revealed the presence of pulmonary venous stenosis in the bilateral lower lobes and middle lobe of patients with bronchiectasis, a discovery proposed for the first time. In comparison to diffuse cases, localized bronchiectasis exhibits a significantly higher incidence of PVS in cases involving left lower lobes (73.5%). Typically, their lesions are confined to one lobe but tend to be more severe. This observation suggests a potential association between this phenomenon and various pathologic changes and clinical symptoms of bronchiectasis.

Pulmonary vein stenosis is an uncommon disease, and there is a remarkable diversity of pulmonary vein connections into the left atrium [27,28,29,30,31,32]. To elucidate the pathophysiological changes observed in cases of bronchiectasis with pulmonary venous stenosis, it is imperative to understand that both the pulmonary and bronchial circulations drain via the pulmonary veins into the left atrium. Consequently, when the drainage of pulmonary veins becomes obstructed, a series of common consequences ensues gradually, including alveolar hemorrhage, compensatory dilation of bronchial arteries, interstitial pulmonary edema, and reduced lymphatic drainage. These pathophysiological alterations may further manifest as hemoptysis, pulmonary infections, and interlobular septal thickening, which are characteristic symptoms of bronchiectasis [9,17,33,34,35,36]. In certain circumstances, pulmonary vein stenosis (PVS) may indirectly contribute to the development of bronchiectasis. When PVS leads to obstructed pulmonary blood flow, it can cause an increase in alveolar capillary pressure, resulting in pulmonary interstitial edema and fibrosis. These pathological changes lead to ventilation–perfusion mismatch, reducing oxygen exchange efficiency and increasing pulmonary vascular resistance [17,37]. With the reduction of blood flow to lung tissue, oxidative stress and hypoxia in the airways are induced, leading to the gradual degradation of airway structures and an increased risk of bronchiectasis [4,38].

Although we cannot definitively establish a causal relationship between bronchiectasis and pulmonary vein stenosis, it is evident that both conditions occur simultaneously in localized bronchiectasis. A study on the outcomes of pulmonary vein stenosis in childhood suggested that pulmonary vein stenosis would result in pulmonary edema, pulmonary hypertension, and potentially cardiac failure, which may not attract much attention until overt manifestations, such as increasingly severe dyspnea, hemoptysis, and recurrent pneumonia [39,40]. Reviewing the CT data of pediatric patients with recurrent lung infections may yield further insights.

According to recent literature, interventional treatments, conventional or sutureless venoplasty, and medical therapies have been reported as effective treatments for pulmonary vein stenosis [40,41,42]. Surgical treatment has been proven effective for eliminating pulmonary vein angulation and straightening the pulmonary vein course, typically involving fibrotic scar tissue extension. Furthermore, balloon angioplasty (BA) and stent implantation have been widely applied to relieve PVS. Compared with BA, stenting was associated with a lower risk of restenosis and required fewer reinterventions. These interventions could potentially be applied in the early stages of bronchiectasis to impede disease progression.

Furthermore, while numerous cases have been reported of patients experiencing severe hemoptysis attributed to pulmonary venous obstruction, our data have not indicated an association between pulmonary vein stenosis and hemoptysis. However, it is plausible that we require a larger number of bronchiectasis cases underwent surgery to be analyzed due to the insufficient sample size.

Our findings indicate that pulmonary venous stenosis, particularly in the left lower lobe, is common among bronchiectasis patients, potentially compounding disease severity and impacting lung volume and respiratory function. Future research should investigate the underlying pathophysiologic mechanisms of bronchiectasis and PVS, the potential benefits of early PVS detection, and whether interventional therapies, such as balloon angioplasty or stenting, may alleviate severe symptoms. Additionally, while our study identified significant associations between PVS and bronchiectasis, the causal relationship remains unclear and requires longitudinal studies to assess progression. A limitation of our study is the relatively small sample size and its single-center nature, which may limit generalizability. Secondly, different scanners and protocols were used for the imaging data of patients at different time periods, and there may be differences in imaging quality. In order to validate and refine these results, a larger multicenter study is necessary.

## 5. Conclusions

Based on our recent findings, pulmonary venous stenosis appears to be a common occurrence in bronchiectasis, often coinciding with reduced lung volume. Specifically, the left lower lobe emerges as a primary site of involvement. Moreover, localized cases are more likely to experience pulmonary venous stenosis, which shows an ambiguous downtrend as the disease advances. Consequently, it is imperative to increase our focus on pulmonary venous variation in bronchiectasis and explore novel therapeutic approaches aimed at early intervention or alleviating the obstruction.

## Figures and Tables

**Table 1:** Demographics and Preoperative Pulmonary Function of Patients with Bronchiectasis vs. Healthy Individuals.

	Bronchiectasis (*n* = 174)	Healthy Individuals (*n* = 91)	*p*-Value
Age, median (range), years	56 (21–85)	57 (29–72)	0.935
Gender, male (%)	72 (41.4)	23 (30.7)	0.110
BMI, M ± SD, kg/m^2^	22.94 ± 3.24	23.69 ± 2.95	0.085
Smoking, n (%)	37 (21.3)	11 (14.7)	0.226
Hypertension, n (%)	47 (27.0)	20 (26.7)	0.955
Diabetes, n (%)	19 (10.9)	8 (10.7)	0.953
Pulmonary Function			
FEV1, median (range), L	2.35 (0.65–4.25)	2.57 (1.24–4.83)	0.001
FEV1, median (range), %,	90.40 (28.70–130.90)	107.20 (61.60–143.40)	<0.001
FVC, median (range), L	3.10 (1.38–5.11)	3.33 (1.86–6.10)	0.015
FEV1/FVC, median(range), %	75.50 (38.70–166.80)	78.45 (56.81–144.40)	<0.001

BMI: body mass index, FEV1: forced expiratory volume in 1st second, FVC: forced vital capacity, M ± SD: mean ± standard deviation. The bold values indicate a value of *p* < 0.05, which is statistically significant.

**Table 2:** Pulmonary vein cross-sectional areas and lung lobe volumes of patients with bronchiectasis vs. healthy individuals.

	Bronchiectasis	Health Individuals	*p*-Value
The Left Upper Lobe			
	*n* = 55	*n* = 50	
PVCA, mean ± SD, mm^2^	149.94 ± 54.15	168.73 ± 50.56	0.070
Lung Volume, M ± SD, L	1.00 ± 0.37	1.00 ± 0.31	0.990
Volume Ratio, median (range), %	0.24 (0.11–0.36)	0.24 (0.10–0.32)	0.259
The Left Lower Lobe			
	*n* = 106	*n* = 66	
PVCA, median (range), mm^2^	83.08 (7.34–189.56)	123.99 (55.37–283.30)	<0.001
Lung Volume, M ± SD, L	0.76 ± 0.33	0.87 (0.27–1.82)	0.003
Volume Ratio, median (range), %	0.18 ± 0.06	0.22 ± 0.03	<0.001
The Right Upper Lobe			
	*n* = 36	*n* = 49	
PVCA, median (range), mm^2^	105.44 (49.77–221.21)	98.43 (51.96–247.39)	0.929
Lung Volume, median (range), L	0.71 (0.36–1.49)	0.82 (0.39–1.40)	0.379
Volume Ratio, median (range), %	0.19 ± 0.06	0.20 ± 0.04	0.134
The Right Middle Lobe			
	*n* = 63	*n* = 64	
PVCA, median (range), mm^2^	22.67 (7.62–94.20)	32.08 (7.02–112.12)	0.009
Lung Volume, median (range), L	0.34 (0.07–0.94)	0.38 (0.15–0.83)	0.066
Volume Ratio, median (range), %	0.08 (0.01–0.20)	0.09 (0.06–0.18)	0.003
The Right Lower Lobe			
	*n* = 54	*n* = 71	
PVCA, median (range), mm^2^	124.12 (31.54–227.14)	137.31 (43.22–347.18)	0.030
Lung Volume, M ± SD, L	0.97 ± 0.33	1.03 ± 0.34	0.366
Volume Ratio, M ± SD, %	0.23 ± 0.06	0.24 ± 0.03	0.493

PVCA: pulmonary vein cross-sectional area, Volume Ratio: lung volume of diseased lung lobe/total lung volume, M ± SD: mean ± standard deviation. The bold values indicate the value of *p* < 0.05, which is statistically significant.

**Table 3:** Different incidence of PVS of localized bronchiectasis and diffuse bronchiectasis.

	Localized Bronchiectasis	Diffuse Bronchiectasis	*p*-Value
The Left Lower Lobe			
	*n* = 49	*n* = 57	
PVS, n (%)	36/49 (73.5)	30/57 (52.6)	0.027
PVCA, M ± SD, mm^2^	71.34 ± 34.83	95.24 ± 44.11	0.003
The Right Middle Lobe			
	*n* = 14	*n* = 50	
PVS, n (%)	10/14 (71.4)	29/50 (58.0)	0.363
PVCA, median (range), mm^2^	21.88 (9.41–49.64)	24.56 (7.62–94.20)	0.661
The Right Lower Lobe			
	*n* = 11	*n* = 43	
PVS, n (%)	5/11 (45.5)	20/43 (46.5)	0.950
PVCA, M ± SD, mm^2^	128.08 ± 47.95	121.09 ± 51.73	0.687

PVS: pulmonary vein stenosis, PVCA: pulmonary vein cross-sectional area, M ± SD: mean ± standard deviation. The bold values indicate a value of p < 0.05, which is statistically significant.

**Table 4:** Preoperative lung function and lung lobe volume of bronchiectasis patients With PVS vs. non-PVS.

	PVS	Non-PVS	*p*-Value
The Left Lower Lobe			
	*n* = 66	*n* = 40	
FEV1, median (range), L	2.34(0.65–3.96)	2.41 (0.85–4.25)	**0.049**
FEV1, median (range), %	90.30 (31.20–124.90)	90.60 (34.20–109.10)	**0.007**
FVC, median (range), L	3.11 (1.38–5.11)	3.11 (1.58–5.08)	**<0.001**
FEV1/FVC, median(range), %	75.06 (38.70–166.80)	74.47 (42.94–85.02)	0.339
Lung volume, M ± SD, L	0.68 ± 0.33	0.88 ± 0.30	**0.002**
Volume ratio, M ± SD, %	0.17 ± 0.06	0.20 ± 0.05	**0.005**
The Right Middle Lobe			
	*n* = 39	*n* = 25	
FEV1, M ± SD, L	2.20 ± 0.83	2.25 ± 0.70	0.844
FEV1, median (range), %	93.84 (28.70–118.20)	93.80 (46.00–110.40)	0.702
FVC, median (range), L	2.84 (1.69–5.08)	3.05 (1.81–4.04)	0.630
FEV1/FVC, median(range), %	75.85 (38.70–96.37)	77.07 (56.88–85.02)	0.364
Lung volume, median(range), L	0.34 (0.07–0.83)	0.34 (0.12–0.94)	0.885
Volume ratio, median(range), %	0.08 (0.01–0.16)	0.08 (0.04–0.20)	0.810
The Right Lower Lobe			
	*n* = 25	*n* = 29	
FEV1, median (range), L	1.89 (1.03–4.25)	2.35 (0.65–3.81)	0.584
FEV1, median (range), %	87.40 (45.70–124.30)	96.70 (31.20–116.30)	0.686
FVC, M ± SD, L	3.10 ± 0.84	3.01 ± 0.91	0.766
FEV1/FVC, M ± SD, %	73.76 ± 10.37	70.94 ± 13.14	0.451
Lung volume, M ± SD, L	0.90 ± 0.33	1.03 ± 0.32	0.149
Volume ratio, M ± SD, %	0.22 ± 0.06	0.25 ± 0.06	0.059

FEV1: forced expiratory volume in 1st second, FVC: forced vital capacity, Volume Ratio: lung volume of diseased lung lobe/total lung volume, M ± SD: mean ± standard deviation. The bold values indicate a value of *p* < 0.05, which is statistically significant.

## Data Availability

The datasets generated and analyzed during this study are not publicly available but can be obtained from the corresponding author and the first author.
